# New Insights in Therapy for Food Allergy

**DOI:** 10.3390/foods10051037

**Published:** 2021-05-10

**Authors:** Cristobalina Mayorga, Francisca Palomares, José A. Cañas, Natalia Pérez-Sánchez, Rafael Núñez, María José Torres, Francisca Gómez

**Affiliations:** 1 Allergy Research Group, Instituto de Investigación Biomédica de Málaga (IBIMA), 29009 Málaga, Spain; francis.palomares@gmail.com (F.P.); joseantonio.canas@ibima.eu (J.A.C.); rafnunser@gmail.com (R.N.); 2 Allergy Clinical Unit, Hospital Regional Universitario de Málaga, 29071 Málaga, Spain; natbel.ps@gmail.com (N.P.-S.); mjtorresj@uma.es (M.J.T.); paquigomez.p@hotmail.com (F.G.); 3 Medicine Department, Universidad de Málaga-UMA, 29071 Málaga, Spain

**Keywords:** food allergy, specific immunotherapy, non-specific therapy, hypoallergens, nanoparticles, microbiota

## Abstract

Food allergy is an increasing problem worldwide, with strict avoidance being classically the only available reliable treatment. The main objective of this review is to cover the latest information about the tools available for the diagnosis and treatment of food allergies. In recent years, many efforts have been made to better understand the humoral and cellular mechanisms involved in food allergy and to improve the strategies for diagnosis and treatment. This review illustrates IgE-mediated food hypersensitivity and provides a current description of the diagnostic strategies and advances in different treatments. Specific immunotherapy, including different routes of administration and new therapeutic approaches, such as hypoallergens and nanoparticles, are discussed in detail. Other treatments, such as biologics and microbiota, are also described. Therefore, we conclude that although important efforts have been made in improving therapies for food allergies, including innovative approaches mainly focusing on efficacy and safety, there is an urgent need to develop a set of basic and clinical results to help in the diagnosis and treatment of food allergies.

## 1. Introduction

Food allergy (FA) is a pathological reaction of the immune system triggered by the ingestion of a food protein antigen. Exposure to very small amounts of allergenic foods can trigger clinical symptoms, such as gastrointestinal disorders, urticaria, and airway inflammation, that range in severity from mild to life-threatening.

The FA prevalence is increasing, and the main treatment consists of food allergens avoidance and in the treatment of the systemic reactions induced by them. New concepts considered as co-factors, environment and nutrition, are changing the epidemiology of allergic reactions to food, leading to new food allergy syndromes. To have an effective treatment, an accurate diagnosis of food allergic patients is required. In this sense, advances in diagnosis are directed to the use of in vivo to in vitro tests. Even so, oral food challenge (OFC) is the only test available to confirm the diagnosis. All these diagnostic approaches could help develop specific treatments in which the relevant allergens causing the reaction should be included. However, further research is urgently needed to achieve this purpose. The development of more precise research has contributed to technological advances, allowing us to understand the mechanisms involved at the cellular and molecular levels that are associated with the concept of immunological tolerance. Currently, the tolerance response that is being achieved using specific-allergen and non-specific-allergen approaches can have some limitations in terms of efficacy and safety, such as the fact that the use of food allergenic extracts from natural sources can cause severe reactions during treatment. As a consequence of this, new prevention models and innovative therapeutic strategies are emerging, aimed at a personalized approach for the patient affected by food allergy.

This review focuses on specific therapies, new therapeutic approaches, and their practical implications in the management of FA, providing an updated view of this complex pathology. This review is relevant because it broadens the knowledge of approaches for a precise diagnosis, for FA management, and for the different alternatives for treatment of FA in both children and adults. This information collects the reduction in the symptoms associated with food allergies and the modulation of the immunological response caused by different therapeutic approaches.

## 2. Food Allergy Epidemiology

There is enough evidence that, over the last decades, food allergy incidence and prevalence have been increasing worldwide, especially in developed countries [1,2]. FA rates have been described to be as high as 10% [3,4,5], and with the absence of a cure for the majority of the types of FA, prevalence will continue to increase. Determining FA frequency in the general population is challenging due to the fact of its complex methodology and to the fact that the data analyzed in most of the epidemiological studies were obtained from different sources including surveys in subjects with self-reported FA [2,3,4,6]. It is more common in children than in adults. Studies based on OFC indicate that the prevalence of FA amongst preschool children is currently between 5% and 10% in some western countries like Australia, and based upon a combination of clinical history and measurement of specific immunoglobulin E (sIgE), nearly 7% in China and Korea [2,4]. To date, in the United States, FA studies are limited to questionnaire surveys, with an FA estimated rate of 5.7% in children in the 2015 National Health Interview Survey, which included data about food allergy in the past 12 months [4] and of 7.6% in another US population-based cross-sectional prevalence survey of over 50,000 households performed between 2015 and 2016 [7].

Regarding FA in adults, a prevalence of 0.3–5.6% was determined in the EuroPrevall project, which also referred to probable FA, considered as the combination of self-reported FA and matching IgE-sensitization. Considering OFC in those cases, the researchers suggest a population prevalence of 0.2–4.1% for confirmed FA. According to the EuroPrevall project, the highest prevalence was found in Switzerland (5.6%), followed by Spain, Poland, the Netherlands, Iceland, and, finally, Greece (0.3%) [6].

FA constitutes an important public health burden in developed countries [1], mainly related to the clinical entities induced by FA, being those induced by an IgE mechanism the most frequent in both children and adults. Although anaphylaxis is a relatively common FA clinical entity, fatal anaphylaxis remains rare, ranging from approximately 0.03 to 0.3 deaths per million persons/year in the general population, and it is most common in young people, with a peak during the second and third decades of life [8,9,10]. Concerning food allergen triggers, in general there are some main allergenic foods which account for 90% of total FA cases: milk, egg, peanut, tree nuts, soya, wheat, fish, and shellfish [6,10,11,12,13]; nevertheless, food triggers also differ depending on country-specific consumption patterns [8].

It is clear that published data show that FA prevalence can vary around the world and that the foods responsible for the reactions differ per region and age. These differences could also be attributed to the development of studies with diverse methodologies, FA definition, environmental factors, such as pollen or dust mites, or even endotoxin exposure [14,15,16,17].

In this review, we describe the different approaches for FA treatment, taking into account that an efficient treatment requires a precise patient phenotyping by studying the mechanisms and diagnosis. This will help to identify the food or allergenic proteins that are actually involved in the reaction and, therefore, be able to prescribe an accurate therapy according to the patient’s needs.

## 3. Mechanisms in FA

FA is defined as an adverse immune reaction to innocuous ingested food proteins or food allergens, which can be mediated by IgE (immediate hypersensitivity reactions), non-IgE mediated, and mixed (immune reactions mediated by IgE and cells) [18].

**Figure 1:**
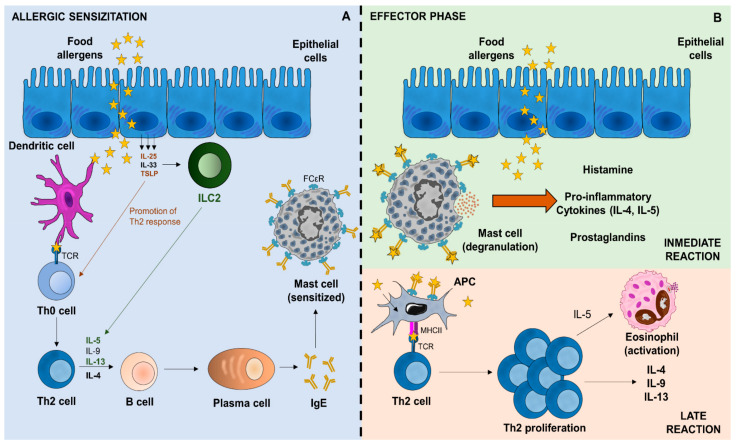
Immunological mechanisms involved in FA: (**A**) Food allergens are taken up by the DC and presented to Th0 cells. ILC2 have been described as IL-5 and IL-13 producers, promoting Th2 response. These cells differentiate into Th2 cells, releasing type-2 cytokines and promoting B cell differentiation into IgE-producing plasma cells. (**B**) Food allergen-sIgE binds to FCεR placed on mast cells. Re-exposure to specific food allergen induces mast cell degranulation and promotes Th2 immune response.

Food allergens, commonly derived from the food proteins of plants or animals, trigger the immune response. They enter the organism by ingestion and can be modified during digestion, being processed by antigen-presenting cells (APCs) and presented on the major histocompatibility complex class II (MHCII) to T cells. Depending on the costimulatory signals, the naive helper T (Th) cells differentiate into Th2 cells in the presence of interleukin (IL)-4, producing a plethora of cytokines such as IL-4, IL-5, IL-10, and IL-13, which induced massive production of sIgE by B cells-derived plasma cells [19]. These allergen-sIgE antibodies bind to the high-affinity IgE receptor (FcεRI) placed on mast cells and basophils (Figure 1A). In addition, epithelial cells produce IL-25, IL-33, and thymic stromal lymphopoietin (TSLP), which can affect the Th2 responses [20]. These cytokines conditioned dendritic cells (DCs) and type 2 innate lymphoid cells (ILC2), leading to expansion and differentiation to Th2 cells [21], which produces Th2 cytokines [22], responsible for allergic processes (Figure 1A). During the successive allergen exposure, sIgE antibodies induce mast cell degranulation, release several pro-inflammatory mediators, including histamine, cytokines, and lipid mediators, promoting the Th2 response that is associated with allergic symptoms (Figure 1B).

## 4. Approaches for a Precise Diagnosis

One of the most challenging issues is the achievement of a precise diagnosis, discriminating between sensitization and real clinically relevant response to foods. Expert panels, practice parameters, systematic reviews, and guidelines have identified several recommended diagnostic modalities [23,24,25]. These tests include medical history, physical examination, elimination diets, skin prick tests (SPTs), sIgE tests, and OFC.

### 4.1. Related to the In Vivo Diagnosis

The most important single “test” for diagnosing an FA is clinical history. This must be reviewed in the context of the clinical manifestations that must be compatible with an allergic reaction, the onset of the appearance of symptoms (time after food intake), the risk factors associated with the patient, the epidemiology of the FA, and keeping in mind disorders with similar clinical manifestations that might be misconstrued. After a well-structured clinical history, testing is probably unnecessary and would likely be confirmatory. Additional diagnostic information is obtained by appropriately selecting and interpreting tests, such as SPTs, sIgE measurements, and OFCs, which must be interpreted within the context of the epidemiology and the pathophysiology [3].

### 4.2. Related to the In Vitro Diagnosis

Classically, the diagnosis of an FA has been performed using allergen extracts containing a mixture of allergenic and non-allergenic molecules that are difficult to standardize. The use of allergen-specific serum IgE (RAST) permits restricting the number of allergen extracts, according to the patient’s clinical history. Thus, diagnosis defines the source but not the allergenic molecule(s) eliciting the disease. In the last decade, advances in molecular allergology have enabled cloning and producing the allergens of major allergenic sources as foods in a recombinant form. This new strategy was deeply integrated in the new precision medicine approach due to the very accurate characterization of the patient’s phenotype [26].

(a) Molecular or component-resolved diagnostic (CRD) tests have been considered promising, and studies regarding their utility continue to emerge. The general premise is that IgE binding to specific proteins in a food might provide more specific diagnostic information than tests that report IgE binding to extracts composed of mixtures of proteins. For example, Ara h 2 is a major peanut protein, a 2S albumin associated with clinical reactions, whereas Ara h 8 is a birch pollen Bet v 1 homolog and is labile and unlikely to be attributable to severe clinical reactions or to be clinically relevant. It is also useful to distinguish different patterns of sensitization related to the geographical area. A Spanish study that included a well-defined group of adult patients allergic to peanut, hazelnut, and walnut concluded that the lipid transfer protein (LTP) was the main allergen in nut allergy [27]. One interesting point related to the CRD is that, for example, an isolated positive result to Ara h 8 would usually suggest general tolerance, since detection of Ara h 2 is related to severe reactions [28,29].

The allergological work up could include the in vitro CRD singleplex tests with extract-based analytes, which are commonly prescribed as a sort of confirmatory evaluation of the in vivo testing, and the single components are performed for an in-depth analysis. Several multiplex systems have recently been developed, such as the ISAC, allowing the evaluation of hundreds of distinct components at the same time and in the same patient. Such an in vitro test could detect a comprehensive profile of IgE sensitization [30]. This tool detects IgE-related sensitization to panels of allergens and gives a more precise and comprehensive evaluation for an IgE-based epidemiology. This insight brings data for a better understanding of the sensitization process.

(b) Basophil activation test (BAT): In recent years, BAT has emerged as a functional assay to differentiate between sensitization and clinically relevant allergy and has proved to be superior to other diagnostic tests in discriminating between peanut allergy and tolerance, particularly in difficult cases, reducing the need for OFCs. Moreover, different parameters, such as basophil reactivity (percentage of activated basophils) or basophil sensitivity (dose at which basophils can react), reflect the severity of allergic symptoms at which patients react during OFC [31,32]. Currently, it is also considered promising [24], although there are challenges related to its use outside the research setting [33].

For now, the OFC is the only test available to confirm the diagnosis, and it is considered the *gold standard*; however, the tests described above can be used judiciously as previous screening steps to reduce the need for OFCs. The main advantage of this type of test is that it is able to differentiate sensitization profiles, predicting patients with more severe allergies such as peanut allergy due the fact of Ara h 2 sensitization [31,32]. However, these tests can be misleading, due to the broad sensitization profiles that they can provide, and it is very important take into account that they must be interpreted by well-trained personnel to avoid misdiagnosis and restrictive diets that are not necessary, impacting on the patient’s quality of life. Studies on alternative diagnostic methods are underway: (i) evaluation of IgE binding to areas (epitopes) on allergens including affinity of binding [34,35,36]; (ii) additional markers being evaluated include cytokines, Treg cells, and T cell number and function; B cell activity; DNA methylation signatures [37]; (iii) bioinformatics approaches with machine learning technology that takes into consideration multiple variables [38]. These alternative diagnostic methods have the advantage of not putting the patient at risk. However, they may be less sensitive and should be interpreted cautiously.

## 5. Primary Prevention and Management of FA

Currently, the traditional approach for managing FA is based on patient education, strict avoidance of the offending food, and prompt treatment of adverse reactions resulting from accidental exposure. Children and families are provided with patient-specific emergency medication and a management plan on how to treat allergic reactions [23]. Recently, with advances in allergy research, a more active approach to managing FA is being adopted. This approach includes early dietary introduction of potentially allergenic foods, as a means to prevent the development of allergy; actively testing for related allergens, once a specific FA has been identified (anticipatory testing); active monitoring and desensitization to known food allergens and active risk management. Although these approaches have the potential to significantly improve quality of life and reduce the development of further allergies, they may significantly increase the complexity of managing children with FA.

The ideal scenario in actively managing FA would be primary prevention. Over the last two decades, there have been significant changes with respect to infant feeding guidance. Complementary feeding was recommended to be delayed until 6 months, with longer delays for specific allergenic foods such as peanuts [39]. This was based on very limited data and has now been withdrawn. Current valid guidelines, written in 2008, recommend exclusive breastfeeding for 6 months and avoidance of potentially allergenic foods (peanuts, other nuts, seeds, milk, eggs, wheat, fish or shellfish) until 6 months of age with no recommendation for any maternal allergen avoidance during pregnancy or lactation. However, even these data are starting to be challenged, as evidence suggests that early introduction of an allergic food may actually play an important role in the prevention of the development of FA with different foods such as peanut, cow milk, or egg [40,41,42]. Moreover, the Learning Early About Peanut Allergy (LEAP) study evaluated the effect of early peanut consumption on the risk of developing peanut allergy, finding an improvement in peanut tolerance in high-risk allergy patients, those with atopic dermatitis and/or egg allergy. The Enquiring About Tolerance (EAT) study is testing the hypothesis that the introduction of six allergenic foods (cow’s milk, egg, wheat, sesame, fish, and peanut) into the diet of infants from 3 months of age, alongside continued breastfeeding, results in a reduced prevalence of FA by 3 years of age. In summary, there are promising results looking at the early introduction of allergenic foods into the diet of infants; however, more rigorous study outcomes are awaited, suggesting that the window of opportunity may vary depending on the food. There are several ongoing interventional studies, which will provide more definitive guidance on introductory feeding for infants.

Recently, anticipatory testing was proposed: the diagnostic process for IgE-mediated FA involves taking an allergy-focused history, followed by targeted allergy testing (either SPTs or sIgE testing), and in cases where diagnostic doubt remains, OFC continues to be the gold standard diagnostic test. Recent FA diagnostic guidance recommends allergy testing to the allergen suspected of causing the index reaction and known co-allergens [43]. It seems that this anticipatory approach has the potential advantages of avoiding unwanted allergic reactions in the community, avoiding unnecessary restrictions in the diet, and also, facilitating early introduction, potentially preventing development of allergy to co-allergens, which requires further investigation. Moreover, this anticipatory approach could have the ability to accurately diagnose FA and differentiate it from sensitization. For example, a Spanish study that evaluated a group of children allergic to nuts describes that sensitization to a particular plant-food LTP did not always cause clinical symptoms with that plant-food. Indeed, 69% (40/58) and 63% (17/27) of peach- and walnut-tolerant subjects had positive rPru p 3- and nJug r 3-sIgE, respectively. Similarly, 9.1% (18/46) hazelnut-tolerant individuals had positive rCor a 8, whereas for peanut it was 36.8% (14/38, Ara h 9^+^) and for wheat it was 26.2% (33/126, rTria 14^+^). Therefore, sIgE and SPTs without a supporting clinical history cannot be used in isolation to definitively diagnose FA [44]. However, for now, an OFC may be required to make a definitive diagnosis, and despite the ongoing improvements in diagnostics, anticipatory testing will inevitably lead to diagnostic uncertainty. As a result, numerous food challenges are likely to be required to make a definitive diagnosis when a patient with no history of consumption is identified as having evidence of sensitization. The economic impact of this will require further study. However, this approach will allow a more precise and prompter diagnosis, the prevention of reactions with potential severe consequences, and the prevention of unnecessary avoidance of related foods [3].

## 6. Allergen-Specific Treatments

FA patients remain asymptomatic when avoiding the ingestion of the food involved. This implies that the only available treatment is the prescription of a restrictive diet that clearly worsens the quality of life of the patient and may even be unnecessary. However, FA patients can develop serious reactions due to the inadvertent and involuntary intake of food. Therefore, there is a need to develop safe and long-term effective therapies [45,46]. In this sense, specific immunotherapy (sIT) in which controlled quantities of an allergen are given to a patient in order to induce tolerance has been proposed as a reliable alternative approach for FA treatment.

### 6.1. Cellular and Molecular Mechanisms

#### 6.1.1. Regulatory and Tolerogenic Mechanisms

The sensitization process starts with the uptake of food allergens or antigens in the gut by special epithelial cells called microfold cells (M cells) and/or goblet-cell-associated antigen passages [47]. In the gut, CX3CR1^+^DCs uptake antigens directly from the lumen and show a higher inflammatory potential than the CD103^+^CX3CR1^−^DC population [48,49]. These last cells have tolerogenic effects and can induce tolerance [50]. After first contact with the allergen, CD103^+^CX3CR1^−^DCs can migrate to mesenteric lymph nodes in a CCR7-dependent manner [51], promoting the proliferation of T regulatory (Treg) cells by means of transforming growth factor beta (TGF-β) and retinaldehyde dehydrogenase 2 (RALDH2) [52]. Therefore, migration of CD103^+^DCs is necessary for induction of tolerance. Moreover, it is very important how the antigen is uptaken to produce tolerance, which has to be through CD103^+^CX3CR1^−^DCs.

Treg cells play a key role in oral tolerance and are essential for modulation of the immunological response [53]. They express CD25 and the factor forkhead box protein 3 (FOXP3) [19]. Indeed, this factor has been shown to be differentially expressed in FA. In fact, children with FA showed lower FOXP3 expression and Treg cell percentage after allergen exposure compared to healthy controls [54,55]. Furthermore, immunosuppressive regulatory B (Breg) cells can also regulate inflammatory response through production of suppressor cytokines such as IL-10, IL-35, and TGF-β [56]. The overexpression of IL-10 by B cells avoids the maturation of DCs, Th2 cell proliferation, and IgE production [57]. In spite of the Breg cells role having been studied in several allergies [58], few human studies have been reported about Breg cells’ responses in FA immunotherapy.

#### 6.1.2. Cellular and Molecular Mechanisms Involved in Immunotherapy

**Figure 2:**
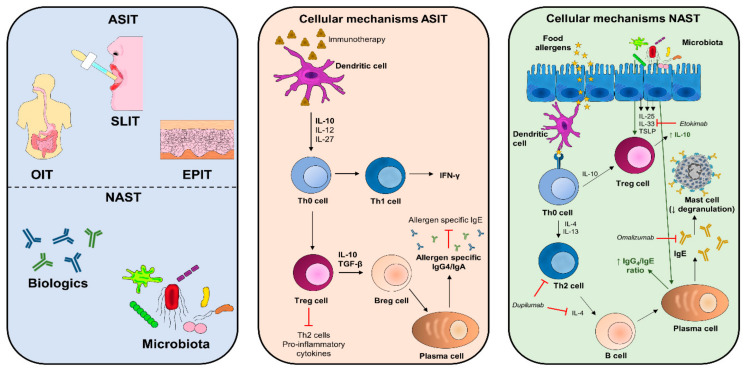
Cellular mechanisms involved in allergen-specific immunotherapy (ASIT) and non-allergen-specific therapy (NAIT). Different therapies exist in the ASIT, such as OIT, SLIT, and EPIT, and in the NAST such as biologics and microbiota. The cellular mechanism in the ASIT implies low doses of food allergen favoring regulatory responses, with Treg cell proliferation, an immunosuppressive Th2 cytokine production, and an immune activation of the Th1 cytokine production to restore the Th1/Th2 balance. All this decreases the production of allergen-sIgE. On the other hand, NAST using biologics directly blocks IgE (omalizumab) or cytokines involved in the Th2 response (dupilumab and etokimab), avoiding cell signaling and all underlying processes involved in FA. In the same way, microbiota acts on the Treg cells by favoring the release of immunosuppressive cytokines (IL-10) as well as plasma cells increasing the immunoglobulin G (IgG)_4_/IgE ratio.

Oral immunotherapy (OIT), sublingual immunotherapy (SLIT), and epicutaneous immunotherapy (EPIT) are the current immunotherapy approaches for FA treatment. Although the underlying cellular and molecular mechanisms involved are not yet fully understood, the most widespread hypothesis is that proliferation of Treg cells and release of immunosuppressive cytokines such as IL-10 occur [59]. Moreover, IT-mediated desensitization is thought to be caused by repeated low doses of antigen exposure during IT treatment, which causes depletion of basophils and mast cells, leading to a reduction of IgE levels [60] that could be associated with a Treg proliferation producers of IL-10 and TGF-β [61] and with Th2 profile shifts to Th1, increasing interferon gamma (IFNγ) production and decreasing that of IL-4 and IL-13 (Figure 2). On the other hand, GATA3 hypermethylation in Th2 cells and hypomethylation of FOXP3 in Treg cells have been discovered in IT-treated mice sensitized to peanut proteins [62].

### 6.2. Specific Types of Treatments According to Types of Foodstuffs

FA-immunotherapy (FA-IT) is an allergen-specific approach to FA handling based on progressively increasing a particular food allergen until reaching a daily maintenance dosage to achieve desensitization [63,64]. Indeed, allergen immunotherapy is currently introduced into clinical practice and it is accepted worldwide [65,66]. However, there are still many unknown aspects which have to be resolved such as the achievement of persistent tolerance [67] and others related to management. In addition, the current coronavirus pandemic (COVID-19) situation must be taken into account, and some reports advise discontinuation of immunotherapy in children infected with severe acute respiratory syndrome coronavirus 2 virus (SARS-CoV-2), because intercurrent viral infections could act as triggers of mild adverse effects or systemic allergic reactions [68]. In pediatric patients suspected of infection as well as in subjects who have recovered from the COVID-19 disease, continuing immunotherapy is recommended but with reduction of the current maintenance dose [69]. However, more studies are needed to determine the efficacy of this treatment. Thus, in this part of review we address the different types of treatments according to food allergens (Table 1).

#### 6.2.1. AIT for Plant-Food Allergy

Hazelnut allergy is the major cause of FA in Europe; therefore, developing therapy is essential for management. Ten allergenic molecules from hazelnuts have been identified and characterized [92]. However, Cor a 9 and Cor a 14 are highly specific for primary hazelnut allergy and strongly associated with severe reactions [93]. A recent study, performed by Morally and coworkers, on children younger than 18 years affected by hazelnut allergy demonstrated that OIT protected against this specific allergy [70]. In this retrospective study, they observed that more than 30% of patients reached desensitization to hazelnut after six months of OIT. Moreover, they reported that this therapeutic approach was safe for patients, with no adverse events associated with hazelnut OIT.

Peanut allergy is often lifelong, persisting into adulthood in 80% of cases, and in the US, it affects up to 2.2% of children and 1.8% of adults [7,94]. The major peanut allergens recognized by the US population are Ara h 1, Ara h 2, and Ara h 3, which are often associated with severe symptoms. However, in Spain, patients recognized these peanut allergens less and were more often sensitized to Ara h 9 [95]. Fortunately, for peanut allergy there are emerging therapies and approved drugs to treat this disorder [96,97], which are described below. The OIT, SLIT, and EPI for the treatment of peanut allergy have been studied in clinical trials with promising results in terms of efficacy [77,81]. A systematic review reported by Chu et al. in 2019 showed that patients undergoing peanut OIT had higher anaphylaxis risk and frequency and achieved a modest degree of desensitization than participants receiving treatment with peanut [71]. Nevertheless, an OIT for peanut allergy, named Palforzia, has been the first approved drug by the US Food and Drug Administration (FDA) in 2020 for patients aged 4 through 17 years with a confirmed diagnosis of peanut allergy [72]. Moreover, other programs for walnut allergies have been announced [98]; however, research continues for the optimization of FA OIT in terms of efficacy, safety, and patient’s quality of life. Regarding peanut allergy and EPIT, a study evaluated the safety and its immunological effects [81]. The results obtained showed that EPIT was safe and associated with a moderate response to treatment after 52 weeks, with a high adherence rate and significant changes in the immunological response (an increase in peanut-specific IgG_4_ levels and IgG_4_/IgE ratios in peanut EPIT-treated participants, along with trends toward reduced basophil activation and peanut-specific Th2 cytokines). Another study performed with Viaskin Peanut (patch) in EPIT, which is under revision by the US FDA, has shown clinical effectiveness [99]. However, the efficacy and safety of these therapies continue under revision.

LTP allergy is frequently associated with other allergies, such as peanut, through LTP allergens such as Pru p 3 (peach allergen) and Ara h 9 (peanut allergen). However, the only treatment that is commercially available to induce a tolerance response to this disease is specific immunotherapy to Pru p 3, the main allergen in peach. Over the last years, several studies have assessed how specific SLIT to Pru p 3 could increase tolerance to peach but also to peanut [79]. The results obtained showed that after 1 year of SLIT, the weal area in SPTs significantly decreased and a significant increase in peach and peanut threshold in treated patients was observed. In addition, immunological changes were reported in only treated patients, with a significant decrease in sIgE and a parallel increase in sIgG4, sIgG_4_/sIgE and basophil reactivity for both Pru p 3 and Ara h 9. In addition, the successful Pru p 3-SLIT was linked to an important immunosuppression of allergen-specific effector Th2 and ILC2, potentially due to the increase in allergen-specific Treg cells. These cellular changes were orchestrated by the activity of DCs promoting the expression of programmed death-ligand 1 (PD-L1) that participates in the regulatory response [59,80]. Currently, this therapy is only available in Spain; thus, the results are limited to a very specific population and might not translate to other countries where Pru p 3 may not be the primary sensitizing allergen [100].

#### 6.2.2. AIT for Animal-Food Allergy

Milk, fish, and egg constitute essential food for children and adults, and restrictions on diet could cause food disorders and a decrease in the quality of life. So, OIT could be a new therapeutic option for cow’s milk allergy, although determination of efficacy is needed. Recently, the risk of severe side effects associated with OIT, such as anaphylaxis and eosinophilic esophagitis, has made it a non-recommended standard treatment [101]. The OIT in children with severe cow’s milk allergy (ORIMA) study, performed by Maeda et al., reported that the treatment efficacy for desensitization was 50%, but the incidence of adverse events was high in 3–13 year old children with a severe cow’s milk allergy [73]. Afterwards, another study demonstrated that continued fixed low-dose OIT yields immunologic improvement and could be effective and safe for severe cow’s milk allergy in children [74], showing the efficacy of OIT at a low dose.

Hypersensitivity to chicken egg is one of the most prevalent FA in children worldwide. The major allergens for infants are found in egg whites, and allergens in egg yolk are less allergenic [102]. To treat this disorder, OIT and SLIT were checked. Recently, a study demonstrated the efficacy of OIT after 8 months with a maintenance dose of 1 g of egg white protein in children with a persistent egg allergy [75]. Likewise, a recent report, performed by Sagara et al., showed two children with egg allergy who were safely treated with SLIT, before transitioning to OIT, who did not have adverse reactions or show mild symptoms such as oral cavity itchiness [78].

In addition, fish is a high-priced source of healthy nutrients; however, 0.1–0.4% of the worldwide population have a fish allergy. Parvalbumin constitutes the main fish allergen, although enolase, aldolase, vitellogenin, and tropomyosin have also been described as allergens [103]. Recently, a Spanish group has reported the use of OIT to hake in several pediatric patients (aged 4–14 years) [76]. They proposed a novel and original protocol based on OIT with a known concentration of protein content and parvalbumin, administrating increasing quantities of lyophilized hake extract, up to reach the target dose of 225 mg. Mild to moderate adverse reactions were recorded during the OIT process, but only antihistaminic and oral corticosteroid treatment was needed to control them. However, further studies on maintenance, efficacy, and safety are needed.

One of the main limitations of using ASIT based on whole extract is the risk of inducing allergic reactions during the treatment because of the use of complete proteins. Moreover, it could also be affected by the lack of standardization of the extract, which will hamper obtaining homogeneous extracts from natural sources in order to guarantee a reproducible, effective, and safe dosage. This is especially critical when treating patients with severe reactions [53,104].

## 7. New Therapeutic Approaches

### 7.1. Hypoallergens

The change or modification of allergens, through a physical or chemical alteration of their structures or selecting the reactive epitopes, aims to improve the tolerance response while preventing allergenicity but maintaining the immunogenicity to induce Th1 and Treg responses. Within the modified allergens, both synthetic peptides and recombinant proteins designed to immunotherapy or vaccines to FA can be found (Table 2).

The use of peptide immunotherapy in FA has been widely explored in a low extent. A phase 1 Australian trial investigating the safety and tolerability of PVX108 (a solution comprised of T cell epitopes of peanut allergens Ara h 1 and Ara h 2) in peanut-allergic adults is currently underway with results still pending (ACTRN12617000692336). Another study identified thirty-six candidate Ara h 1 T cell epitopes 1 with the capacity to stimulate T cell proliferation in peanut-allergic patients compared to non-allergic controls. From them, a mix of seven peptides was associated with the strongest T cell responses and was used in basophil degranulation assays utilizing whole blood from peanut-allergic patients. There was no evidence of degranulation at any of the three tested concentrations, suggesting that these peptides had no IgE cross-linking capabilities [117] and could protect from the anaphylaxis reaction.

Meanwhile, allergen T cell epitopes are preserved, which allows IgG antibody production and promotion of the regulatory and Th1 response. Clinical trials are testing the safety and efficacy of the use of recombinant allergens in immunotherapy in the treatment of FA. A phase 1 trial studied the effects of recombinant modified peanut proteins Ara h 1, Ara h 2, and Ara h 3 using an Escherichia coli vector. This study found a change in the immunological profile: from Th2 towards Th1 profile; this happened without a significant change in peanut-specific IgE or IgG levels in peanut-allergic patients [118]. Other hypoallergens have been studied for fish allergy and apple allergy. The use of the hypoallergenic carp parvalbumin mutant could allow treating fish allergy in allergic patients [119]. Recently, another study showed that the recombinant Mal d 1 combined with immunotherapy blocked the IgE mediated reactions, downregulated the allergen-specific Th2 response [105] improving apple allergy [106]. However, another study using sera from peach allergic patients and a recombinant Pru p 3 showed a decrease in IgE levels with a mild basophil activation confirming the no allergenic potency of the recombinant compared to native Pru p 3 [120]. Despite these results, a recent study showed that the combination of immunotherapy with native allergen of Pru p 3 had more beneficial effects (production of IL-10 and IFN-γ) than the hypoallergenic variant of Pru p 3 in an animal model [121].

Based on these findings, the approach of using recombinant allergens, in theory, enables the avoidance of potential immune reactions due to the ablation of IgE binding and, thus, downregulation of downstream IgE effects. One of the possible limitations of using peptides is the lower immunological response; therefore, further study should be considered.

### 7.2. Nanoparticles

Nanoparticle delivery systems are in the process of being developed with properties to enable reduced degradation in the intestinal tract, increase uptake efficiency, and modulate the immunological response [122]. The polymeric biodegradable nanoparticles, such as polyesters (synthetic), polysaccharides, and polyamides (natural), and virus-like particles allow encapsulation of allergens or protein allowing uptake into APC without IgE binding [123,124] (Table 2). The nanoparticles containing allergen can be coated with adjuvant [125], such as cytosine-phosphate-guanine (CpG) oligodeoxynucleotides (ODNs), or carbohydrates to promote Th1/Treg responses (Table 2).

Recently, different studies have shown that the T cell epitope of the shrimp allergen arginine kinase (AKp) with TLR9 agonist CpG-ODNs in nanoparticles reduced the allergic response. This treatment demonstrated a reduction in anaphylaxis reactions with a decrease in sIgE levels and increased FOXP3 and IL-10 expression in shrimp allergic mice [107]. In addition, in a murine model of peanut allergy, the CpG-coated poly (lactic-co-glycolic acid) (PLGA) nanoparticles containing peanut extract were shown to decrease levels of Th2 cytokines and increase IFN-γ levels, indicating that peanut immunotherapy with CpG/PN-NPs might be a valuable strategy for peanut-specific immunotherapy in humans [126]. Another study investigated the immune response induced by protamine-based nanoparticles (proticles) with CpG-ODNS as allergen delivery system. These nanoparticles complexed with Ara h 2 extracted from peanuts were assayed in mice model and they counteracted the Th2 immune response induced by an allergen [127]. Similar results were found for peach allergy in which the immunotherapy based on systems containing dendrimers with Pru p 3 T cell peptides and CpG–ODNs induced a Th1/Treg response in peach allergic mice [108], decreasing anaphylactic reactions.

Currently, a new approach focuses on the modulation of the immune response via C-type lectin receptors (CLRs) using specific nanoparticles such as the glycosystems functionalized with mannose. Regarding this, glycosylated nanostructures with Pru p 3 peptides combined with immunotherapy induced long-lasting tolerance in a peach allergy mouse model. This work showed an increase in Treg cells and regulatory cytokines (IL-10^+^/IFN-γ^+^) in CD4+T-cells and DCs [109]. Furthermore, the lower concentration used for the glycosylated nanostructures with Pru p 3 peptides induced a tolerance response, while the higher concentration induced desensitization to peach [109]. In addition, these Pru p 3 peptide glycosystems promoted changes in the properties of DCs and T Cells and did not activate the basophils from peach allergic patients [110].

Other nanostructures of different chemical compositions, such as poly (anhydride) nanoparticles, have been studied in nut allergy. In fact, the poly (anhydride) nanoparticles containing nut proteins could induce a strong Th1 and Treg immune response after oral administration in a murine model of fatal anaphylaxis [111,112,113]. In addition, mannosylated nanoparticles for OIT in a murine model of peanut allergy showed both less serious anaphylaxis symptoms and higher survival rates than the control group, confirming the protective effect of this formulation against the challenge [114]. PLGA nanoparticles and beta-lactoglobulin (BLG)-derived peptides facilitated oral tolerance preventing cow’s milk allergy in mice [115]. However, the chemical design of these structures must be taken into account, since there are different pathways by which the immune response can be modulated in FA, and more studies are needed to elucidate whether the nanostructures are immunostimulatory in mice and humans.

Recently, a research group developed an immunogenic, protective, and non-reactogenic vaccine candidate against peanut allergy based on virus-like particles (VLPs) coupled to single peanut allergens. They generated vaccines with extracts of roasted peanut (Ara R) or the single allergens Ara h 1 or Ara h 2 coupled to Cucumber Mosaic Virus-derived VLPs (CuMVtt). The mice were sensitized to peanut extract and the immunotherapy consisted of a single subcutaneous injection of CuMVtt coupled to Ara R, Ara h 1, or Ara h 2. The vaccines CuMVtt-Ara R, CuMVtt-Ara h 1, and CuMVtt-Ara h 2 protected peanut-sensitized mice against anaphylaxis after intravenous challenge with the whole peanut extract. From them, CuMVtt-Ara h 1 was able to induce sIgG antibodies, diminished local reactions after SPTs, and reduced the infiltration of the gastrointestinal tract by eosinophils and mast cells after oral challenge with peanut [116].

## 8. Non-Allergen-Specific Therapy

### 8.1. Biologics

Recent studies have begun to evaluate the use of biologic agents in the treatment for FA. The majority of these biologics are characterized by their ability to inhibit and suppress the pro-inflammatory pathways specific to FA (Figure 2). In fact, they are commonly used in monotherapies as adjuncts to ASIT in order to increase safety and prevent the food allergic reaction (Table 1).

Omalizumab, a humanized anti-IgE monoclonal IgG_1_ antibody, is one of the most widely researched biologics in the treatment of IgE-mediated FA. It has been studied both as a monotherapy [82] and as an adjunct to milk-, egg- or peanut-OIT, demonstrating an increase in the safety and efficacy compared to placebo in food allergic patients [83]. It has also been studied in a number of multi-allergens OIT trials in which omalizumab together with OIT decreased time to achieve desensitization and rapidly desensitized to multiple food allergens, improving the efficacy of multi-food OIT [84,85]. At the cellular level, patients treated with omalizumab and milk-OIT, without interruption of treatment, showed alterations in basophil reactivity with a low CD63 expression [86] without affecting Treg levels.

Dupilumab is a recombinant human IgG_4_ monoclonal antibody directed against the α-chain of the IL-4 receptor (IL-4Rα) that is common to both IL-4 and IL-13. The first report of its efficacy in the field of FA was a case report in which a patient with severe atopic dermatitis and FA receiving dupilumab became desensitized and passed a confirmatory OFC to two foods previously responsible for allergic reactions [128]. Currently, two placebo-controlled studies are underway. These studies evaluate the efficacy and safety of the dupilumab monotherapy and as an adjunct to peanut-OIT. All the studies are in phase 2 (NCT03793608 and NCT03682770, respectively).

Other therapeutic targets that are being studied are IL-25, IL-33, and TSLP. They play a critical role in the development and maintenance of FA, and antibodies against them are reported to suppress FA in murine [129]. Etokimab, a humanized IgG_1_κ monoclonal antibody, has been specifically developed to bind to and neutralize the biological effects of human IL-33. There is a phase 2a study that suggests that etokimab is safe and well tolerated, and that it can have in a single dose the potential to desensitize peanut-allergic patients possibly reducing atopy-related adverse events (NCT02920021). However, anti-IL 25 and anti-TSLP therapies are limited in FA, and the few available data are described in animal in vivo and in vitro models [130,131].

### 8.2. Microbiota

The relationship between the state of the intestinal microbiota and FA is being deeply investigated. Several studies are focused on searching microbial therapies that can prevent and/or treat FA. Probiotics, prebiotics, synbiotics, and fecal microbiota transplantation (FMT) represent novel interventions to modulate the gut microbiome toward beneficial outcomes in FA (Table 1).

Probiotics are live microbes, the intake of which is thought to be beneficial for health [132]. Several clinical trials have examined that, in patients with cow’s milk allergy, a diet supplemented with *Lactobacillus casei* or *Bifidobacterium* induced tolerance to cow’s milk [87]. However, a clinical trial of patients with milk allergy receiving a supplementation with a hydrolyzed casein formula containing a probiotic, *Lactobacillus rhamnosus GG*, reduced the occurrence of allergic manifestations [86]. Moreover, *L. rhamnosus GG* has also been studied as a microbial adjunct to OIT for peanut allergy. In a clinical trial in which the allergic patients were included to receive *L. rhamnosus GG* with peanut OIT showed that the treatment was effective, inducing a modulation of allergic response (smaller peanut SPTs response size) with immunological changes (higher peanut sIgG_4_/sIgE ratios). However, this work failed to clarify whether these changes were induced by probiotics or OIT [88]. Although the results of some of the previous trials suggest that probiotics could be useful for FA, more studies are needed to determine what their role is in tolerance response development. Moreover, statements from the World Allergy Organization comment that there is limited and “very low-quality evidence” on this topic [133].

Another approach to shaping the gut microbiota is the provision of components that foster the growth of desired microbial community members. This is the idea behind prebiotics, non-digestible food components that specific commensal microbes can use to selectively foster their growth and activity. The prebiotics are oligosaccharides that are not digested or absorbed in the gastrointestinal tract before they reach the large intestine, where specific microbiota can use them for improved growth and activity. Although prebiotic supplementation in infants was found to be associated with decreased risk of asthma and eczema; in some investigations there have been no significant effects on the development of FA found so far [134].

Synbiotics are combinations of probiotics and prebiotics. It is thought that they might be more effective than probiotics or prebiotics alone because the provision of both together delivers a synergistic combination of desired microbes and the components they need to thrive and drive the host community [135]. A prospective study of infants with cow’s milk allergy receiving a synbiotic-containing amino-acid-based formula improved the fecal microbiota of them [89]. In this case, the synbiotics used in this trial included the probiotic *Bifidobacterium breve M-16V* in combination with the prebiotics oligofructose and acidic oligosaccharides. However, more studies are required to confirm this therapeutic effect of synbiotics supplementation in desensitizing FA. It is a promising therapeutic strategy for improving the gut ecosystem and reducing FA responses.

FMT is a possible therapeutic for FA. Transplantation of fecal bacteria from a healthy donor to a disease recipient can re-establish gut microbiota diversity leading to the resolution of symptoms. The studies in murine models to identify the microbiota were the basis for FMT in humans with FA. They showed that FMT from healthy human infants and transplanted into a food-allergic mouse model protected from anaphylaxis after allergen exposure, whereas anaphylaxis was not abrogated when FMT was performed with stools taken from food-allergic infants [136,137]. Other mouse studies, investigating clusters impacted by dysbiosis in infants, utilized a type of mouse, Il4raF709, which is genetically prone to FA, showing that the bacteriotherapy containing commensal *Clostridiales* strains with or without *Bacteroidales* strains both prevented and treated FA. In fact, the bacteriotherapy induced expression by Treg cells in a MyD88/RORγt dependent manner, which suppresses FA in infants and mice [90]. In a different FA mouse model, with another species of Clostridia known as *Anaerostipes caccae*, demonstrated that intestinal bacteria were critical for regulating allergic responses to dietary antigens [91]. The research advances in the microbiome field have moved FA therapy into the arena of microbiome-related human clinical trials. Regarding this, a small phase 1 trial (NCT02960074) is evaluating the safety and tolerability of encapsulated FM, which is administered via oral for the treatment of peanut allergy in adults. In addition, another study (NCT03936998) is determining if VE416 in combination with vancomycin (an antibiotic) could be used as OIT in peanut allergy patients. VE416 is a set of commensal bacteria capsules pre-treated with vancomycin to facilitate the colonization of the transferred bacteria. Although the available data in this field remain limited and the relevant scientific work has just begun, this recent success in reducing infantile allergy colitis symptoms suggests that fecal microbiota transplantation can be a feasible strategy to arrest FA responses.

## 9. Limitations and Conclusions

This review has collected different published studies that show the beneficial effects and limitations to treat FA.

FA is still a growing public health concern worldwide, with a high incidence, and constitutes a public health burden in developed countries. One of the main challenges is the clinical management related to the diagnosis and treatment. In recent years, the efforts have been focused on improving treatment. Despite these efforts, some limitations have been found throughout this review, based on a bibliographic search, associated with last studies in FA treatment. In this regard, specific therapeutic approaches, such as ASIT and NASIT, can have several disadvantages: they require a long-time to build up the immune response, after stopping the treatment, the sensitization to the foods involved can reappear, and they can pose a risk to the patients, as they can have an allergic reaction to the food at any given dose. In addition, concerning the use of biologics for FA treatment, results proving if tolerance response is sustained are required, consensus on how these biologics should be administered is lacking, and, moreover, it is a very expensive therapy. Within NASIT, microbiota also present certain limitations associated with a lack of relevant results and with differences in protocols for assessing sustained unresponsiveness. Even so, these limitations have led to the emergence of new questions that have enabled the development of new treatment tools. These new therapeutic approaches, like the hypoallergens and nanoparticles, are mostly being studied in animal models and in very few cases in humans. Therefore, more studies in humans are needed to address the limitations of these therapeutic approaches in FA.

Despite the limitations described above, the ASIT has the potential to balance the immune dysregulation causing the food allergic inflammation and it has demonstrated its ability to prevent future sensitizations, being safe, effective and inducing food tolerance.

Therefore, it is necessary to develop new therapeutic strategies that can help manage FA. In this sense, recent developments in FA research have focused on new therapeutic approaches such as the use of the hypoallergens and nanostructures. They are designed to be more safe, precise, and faster than immunotherapy. These therapies are currently at different developmental stages. The hypoallergens have demonstrated to be promising as a way to improve safety and enhance efficacy of immunotherapy; but further studies, particularly in human subjects, will be necessary to confirm safety and efficacy. Nanoparticles are being studied since they can enhance the delivery of the food antigen. Theses nanoparticles can stimulate Th1 and/or T regulatory responses avoiding the IgE cross-linking on mast cells and basophils. These properties open new possibilities for treating not only food allergies but also allergic diseases.

On the other hand, NASIT, such as the targets for biological drugs or microbiota, are being developed but none have progressed to the large scale. The use of biologics that function through the inhibition of pathways specific to FA can provide a safe tolerance response to multiple foods. Moreover, the influence of microbiota on the immune system is an exciting area of research and, specifically, the role of probiotics as adjuvants in vaccinations or immunotherapy is very promising.

Summarizing, more research is needed to develop an “ideal” therapy that shows promising efficacy in modulating the immune response, facilitating shorter dose phases (which would increase patient adherence to treatment), reducing adverse reactions during treatment, and finally inducing tolerance to food.

## Figures and Tables

**Table 1:** Summary of the therapeutic approaches for FA in the last 5 years.

Treatment	References	Food	Benefits	Limitations
Allergen-specific therapy				
OIT	[70,71,72]	Hazelnut	Safe desensitization. Robust, possible sustained.	Time-consuming Side effects
		Peanut	Effective desensitization. Palforzia increases the amount of consumed peanut protein.	
	[73,74]	Milk	Improvement and safety in a continued fixed, low dose.	
	[75]	Egg	Effective and a humoral immune response.	
	[76]	Fish	Parvalbumin–OIT consisted of a quick build-up phase. Target dose equivalent to a typical portion of fish.	
SLIT	[77]	Peanut	Clinically meaningful and safe desensitization in children. Minor side effects, brief exposure.	Lower food intake threshold than OIT
	[59,78,79,80]	Egg	Decrease in sIgE levels.	
		Peach	Desensitization and cellular immunological changes.	
EPIT	[81]	Peanut	Increase in the food tolerance in children. Minor side effects.	Less robust than OIT More effective in those younger in age
Non-allergen specific therapy				
Omalizumab (anti-IgE)	[82,83]	Peanut	As monotherapy, threshold increased for food allergens and decreased dietary restrictions. As adjuvant with OIT, rapid oral desensitization. Lower rates of allergic reactions from IT.	No effect on likelihood of developing sustained unresponsiveness Expensive
Dupilumab (anti-IL-4)	[84,85]	Multi-food	As adjuvant with OIT, efficacy improved for multi-food OIT. Safe and rapid desensitization. Faster dose escalation.	
Etokimab (anti-IL-33)	[86]	Milk	As adjuvant with OIT, basophil reactivity modification.	
Probiotic Synbiotic FMT	[87] [88] [89] [90] [91]	Milk Peanut Milk Multi-food Milk	Decrease in the incidence of other allergic manifestations. Hastened the development of oral tolerance in children. Increase in rates of sustained unresponsiveness. Smaller peanut SPTs response size. Efficacy increase. Immunological changes (higher peanut sIgG4/sIgE ratio). Improvement in gut microbiota in allergic infants. Action via regulatory T cell myd88/RORγT pathway to suppress FA. Modulation of bacterial communities.	Lack of relevant results Differences in protocols for evaluating sustained unresponsiveness Requires more studies

EPIT: epicutaneous immunotherapy; FA: food allergy; FMT: fecal microbiota transplantation; IT: immunotherapy; OIT: oral immunotherapy; sIgE: specific IgE; SLIT: sublingual immunotherapy; SPTs: skin prick tests.

**Table 2:** Summary of new current therapies for FA.

Treatment	References	Food	Benefits	Limitations
Recombinant proteins	[105,106]	Apple	SLIT with recombinant Mal d 1 downregulated the allergen-specific Th2 response. Blocking the IgE production. Potentially safe (low allergenicity).	Extensive mapping of T cell epitopes
	[107]	Shrimp	Reduction of a shrimp allergen-induced Th2 response in FA. Protection against anaphylaxis in murine models.	
Nanoparticles with T cells epitopes and/or CpG	[108,109,110]	Peach Nut Peanut	SLIT using nanoparticles with Pru p 3 induced a Th1/Treg response. Increase in Treg cells, IL-10, and IFNγ levels. Changes in the DC and T cells.	Concerns about the release of the profile and potential for burst release of large amounts of allergen at once.
Poly (anhydride) nanoparticles	[111,112,113]	Peanut	Strong Th1 and Treg immune response decreasing anaphylaxis in a murine model. Mast cell levels reduction, increasing survival rate.	
Mannosylated nanoparticles	[114]	Peanut	Reduction of the serious allergic symptoms. Protective effect.	
PLGA nanoparticles	[115]	Milk	Modulation of mucosal immunity. Facilitating milk allergy prevention.	
Virus-like particle	[116]	Peanut	Blocking of the allergic response with a favorable safety profile.	

DC: dendritic cells; FA: food allergy; PLGA: poly (lactic-co-glycolic acid); SLIT: sublingual immunotherapy.

## Data Availability

Not applicable.
